# Digital Health Coaching in Pregnancy: A Scoping Review of Interventions and Outcomes

**DOI:** 10.34172/hpp.45284

**Published:** 2026-08-21

**Authors:** Soheila Jahangiri Mirshekarlou, Leila Nikniaz, Peyman Rezaei Haches, Mohamad Jebraeily, Elham Rajabi, Taha Samad-Soltani

**Affiliations:** ^1^Department of Health Information Technology, Faculty of Management and Medical Informatics, Tabriz University of Medical Sciences, Tabriz, Iran; ^2^Student Research Committee, Tabriz University of Medical Sciences, Tabriz, Iran; ^3^Department of Health Information Technology, School of Allied Medical Sciences, Urmia University of Medical Sciences, Urmia, Iran; ^4^Department of Obstetrics and Gynecology, School of Medicine, Research Institute on Cellular and Molecular Medicine, Kosar Hospital, Urmia University of Medical Sciences, Urmia, Iran

**Keywords:** Digital health, Health coaching, Mentoring, Pregnancy, Motivational interviewing

## Abstract

**Introduction:**

Health coaching is an innovative model of health promotion, empowering individuals to enhance self-care, make informed choices, and develop health knowledge and skills. Integrating digital technologies like mobile apps, web platforms, wearables, and artificial intelligence into health coaching enables personalized, accessible, and continuous support to improve disease management. In maternal health, digital coaching promotes healthy behaviors in nutrition, physical activity, sleep, and mental well-being. This review explores digital health coaching interventions during pregnancy and their impact on maternal and fetal outcomes.

**Methods:**

We conducted a scoping review of English-language articles from 2000 until December 2024 in PubMed, Scopus, Web of Science, IEEE, and Google Scholar. Keywords that formed the search strategy were "digital health"," health coaching ","pregnancy" along with synonyms and related terms.

**Results:**

Thirty- one studies were included. Most interventions used smartphones, text messaging, coaching applications, virtual sessions, and wearable devices focusing on nutrition and physical activity to manage weight and gestational diabetes. Nutritionists, obstetricians, and midwives were the main contributors, using motivational interviewing, goal setting, and social cognitive theories. Outcomes showed acceptability and satisfaction in 45% of studies, feasibility in 35%, and effectiveness in lifestyle behaviors in 68%. Improvements in weight management, glycemic control, and body composition were observed in 42% of studies.10% noted benefits in maternal and newborn health. One study reported cost-effectiveness; however, some found no significant differences in gestational weight gain or diabetes.

**Conclusion:**

Digital health coaching in pregnancy can have favorable potential to support mothers’ health and change their behavior. However, more research is needed on the most effective components, including psychological aspects and socio-economic measures.

## Introduction

 Health coaching has recently become a prominent trend in health promotion, providing a valuable resource for increasing individuals’ awareness and promoting behavioral change. Additionally, it is a potent approach to encourage self-care and adherence to health goals.^1,2^ It involves a collaborative effort between healthcare professionals and individuals, empowering them to actively participate in healthcare decisions, including adhering to exercise routines and dietary choices.^1,3^ Due to eliminating distance and time barriers, coaching strategies incorporating digital technologies such as web-based platforms,^4^ mobile and gamified application,^5,6^ and AI-driven autonomous coaching systems have been increasingly implemented.^7^ The reduced costs and enhanced accessibility have enabled two-way communication between patients and specialists through remote monitoring of lifestyle habits, resulting in effective outcomes.^7,8^ Digital coaching refers to services and interventions that are carried out using technology-based devices or methods that not only provide feedback but also provide recommendations, suggestions, and next steps for the user to pursue their health goals.^4,9^ Consequently, digital health coaching interventions have emerged as a promising strategy in various health domains including the management and prevention of chronic diseases like diabetes, cancer, and mental illnesses, as well as for supporting expectant mothers.^7,8,10-12^ In pregnancy, these models of care have demonstrated clinically and economically effective outcomes.^13,14^ They improve diabetes management and help control gestational weight gain and cholesterol intake.^10,15,16^ They also enhance lifestyle behaviors, including nutrition and physical activity, while increasing health awareness.^17-19^ In addition, they contribute to reducing adverse neonatal outcomes such as respiratory distress and perinatal mortality.^10^ These interventions provide personalized and accessible support throughout pregnancy, while facilitating behavior change and strengthening patient–specialist relationships to improve the delivery of essential maternity care services.^18,20^

 Therefore, considering the importance of digital coaching in pregnancy and the need to understand its key features for successful design and implementation, as well as the lack of a comprehensive overview of existing interventions, this scoping review seeks to address existing gaps. Specifically, as the frameworks, components, and structural models employed have not been clearly or systematically identified in the literature, this review maps the current status of digital health coaching interventions and outcomes in pregnancy. It focuses on their implementation strategies, effectiveness, and reported impact on maternal and fetal health outcomes, including contributions to delivering coaching services and supporting pregnant women.

## Methods

###  Methodological Framework

 This scoping review used the Arksey and O’Malley^21^ framework. Our process is in accordance with its key stages: identifying the research question, conducting a systematic search, selecting relevant studies, charting the data, and extracting and reporting the results. For comprehensive and transparent reportingof this scoping review, we followed the Preferred Reporting Items for Systematic reviews and Meta-Analyses extension for Scoping Reviews (PRISMA-ScR) standard, specifically by using its flow diagram to illustrate the selection process of studies included in this scoping review.^22^ (See Figure 1)

**Figure 1:**
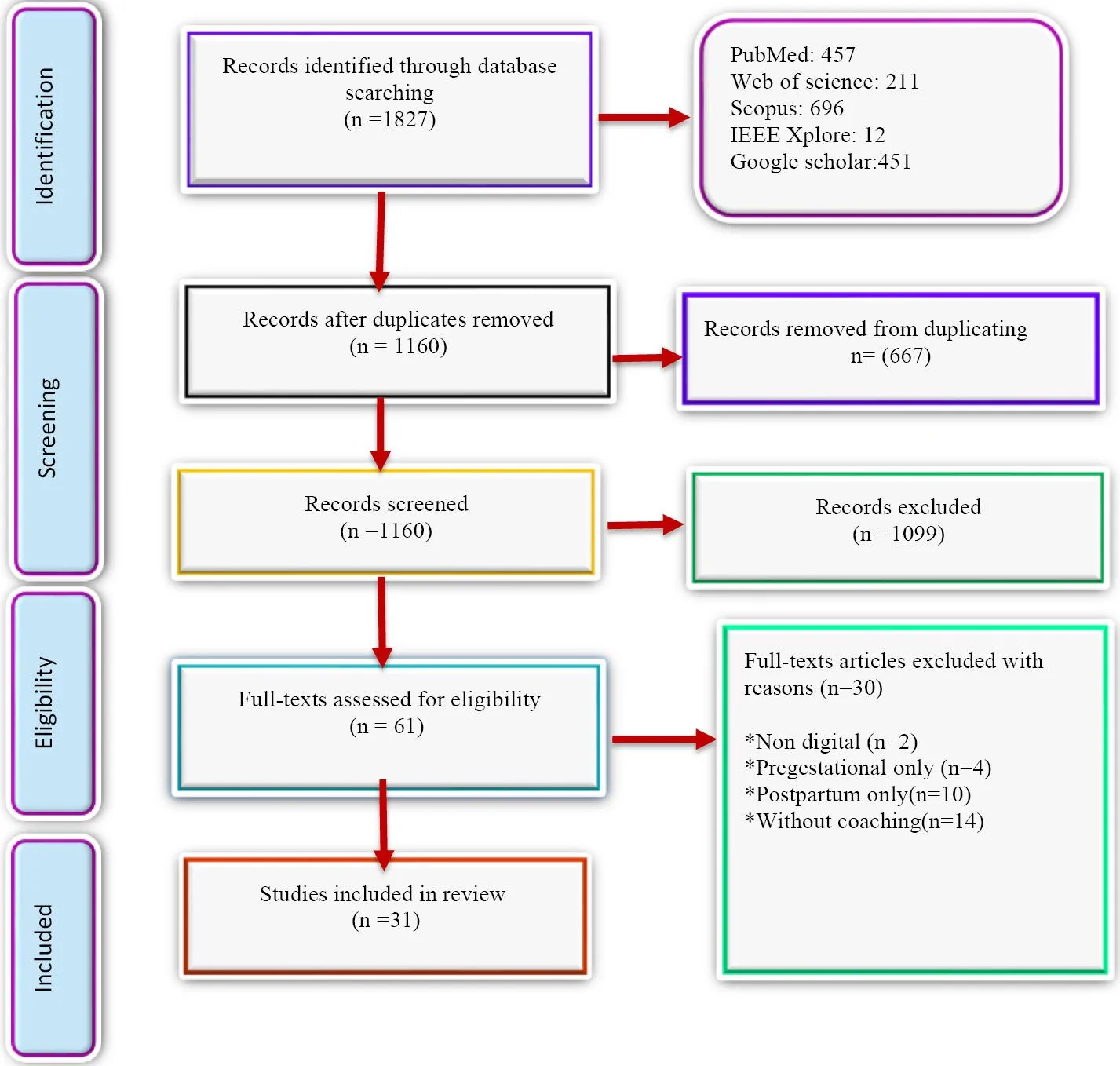
PRISMA-SCR flow diagram of the study selection process for the scoping review on digital health coaching interventions in pregnancy

###  Search Strategy 

 This scoping review was conducted on research studies that provide information about the use and evaluation of digital health coaching interventions in pregnancy. PubMed, Web of Science, IEEE Xplore, Scopus, and Google Scholar databases were searched using a combination of subject headings (MeSH in PubMed) and free-text terms limited to the title, abstract, and keyword fields. The search terms were: (“digital health “OR “Telemedicine “OR “telemedicine” OR “telehealth “OR “e-health” OR “virtual health” OR “mobile health” OR “online health” OR “health app*” OR “video health”) AND (“Mentoring” OR “coach*” OR “health coaching” OR “mentor*” OR “consult*”) AND (“Pregnancy” OR “pregnan*” OR “prenatal” OR “perinatal” OR “antenatal” OR “ante-natal” OR “gestation*” OR “matern*”). The literature search encompassed publications from 2000 until December 2024. Studies were imported into EndNote software, and duplicates were removed. The screening was conducted in three stages: title, title/abstract, and full text, based on the inclusion and exclusion criteria. The screening was performed independently by two reviewers, and any discrepancies were resolved through discussion. The data from the included studies were charted using two data extraction tables. Table 1, titled “study features,” with Author/year of publication, Country, platform/app name, Objective, Study design, Sample size, and intervention time fields. Table 2, titled “Study Technological Features,” included: author/year of publication, coaching practitioner, coaching targets in pregnancy, technology used, coaching methods/theories/app developing, and outcomes.

**Table 1:** Study Features

**Author/year of publication**	**Country**	**Program/app name**	**Objective**	**Study design**	**Sample size**	** Intervention Time**
Preconception +pregnancy						
Van Dijk 2016 ^34^	Netherland	Smarter pregnancy	Assessment of online web-based coaching program effectiveness, usability, and compliance	Survey study	women and men trying to get pregnant1275 and pregnant 603	*Trying to get pregnant *Intervention:6 months
Gootjes 2019 ^17^	Netherlands	Smarter Pregnancy	Investigate the relationship between deprivation & improvement of poor dietary habits and lifestyle behaviors with my health coaching among women who wish to be pregnant or are currently pregnant.	Observational	2554 women, 521 are participating with their male partner	*Pregnant for less than13 weeks alone or with partners *Intervention: 24 weeks
van Dijk 2020 ^35^	Netherlands	Smarter Pregnancy	Determine compliance with the Smarter Pregnancy intervention& empower women to adopt a healthy diet before pregnancy and during early pregnancy	RCT	*218 women and 36 men. -intervention group:(109 women and19 men) - control group: (109 women and17 men)	*Women wanted to get pregnant or were pregnant for less than13 weeks (to 24 weeks after requirement) *Intervention:6 months
pregnancy						
Dodd 2017 ^28^	Australia	SNAPP	Impact assessment of smartphone application on facilitating physical activity & nutrition in pregnant women to provide lifestyle advice	Two RCT (GROW &OPTIMISE)	162(- 77 women in advice lifestyle and application group-85 women in advice life style group) for two trials	*Grow study in10 to 20 weeks *OPTIMISE study for 28 to 36 weeks in pregnancy intervention: until 36weeks
Jelsma 2017 ^33^	Europe (Austria, Belgium, Denmark, Ireland, Italy, Netherlands, Poland, Spain, United Kingdom)	DALI	Assessment of lifestyle intervention based on motivational interviewing for GDM Prevention in obese pregnant women	Cross-sectional	144 pregnant *3 intervention groups: -healthy eating (HE,48), -physical activity (PA,47), -HE and PA (HE + PA,49)	*Before 20 weeks of gestation *Intervention: until 35 -37weeks of gestation:
Willcox 2017 ^29^	Australia	txt4two	Promoting a healthy diet, physical activity, and GWG within the IOM guidelines using multi mHealth modalities	RCT pilot study	100 pregnant women (being obese or overweight)	*Between 10 and 17 weeks of pregnancy *Intervention: from the first visit of pregnancy to 36 weeks’ gestation. (follow-up in 36 weeks)
Seward 2018 ^19^	USA	-	Evaluation and acceptability of health coaching intervention remotely to have healthy lifestyle behavior & suitable GWG in obese pregnant mothers	Pilot study	30 overweight pregnant women	* < 16 weeks pregnant *Intervention: until 36 weeks
Sung 2019 ^36^	Korea	Huraypositive	Develop and evaluate a model for the management of GDM with the use of mobile healthcare.	RCT -pilot study	21 pregnant women with GDM *Intervention group:11 *control group:10	*24-28 weeks pregnant *Intervention: until 37 weeks of pregnancy evaluation after delivery
Li 2019 ^16^	Singapore	Glycoleap	Testing the food coaching application for optimal gestation weight (GWG) gain in obese pregnant mothers who have type 2 diabetes& improving care for them.	RCT pilot study	30 overweight women (15 in every group)	*18-20 weeks of pregnancy-*intervention: 8 weeks
Darvall 2020 ^30^	Australia	Fit mum study	Access to a pedometer intervention to increase activity and decreasing excessive GWG in obese pregnant women.	RCT	30 women in two interventions and one control group (10 in every group)	*12-16 Weeks of Pregnancy * Intervention:10 weeks
Yee 2020 ^27^	United States	Texting for Diabetes Success (TDS)	Text messaging program development and evaluation for diabetes management in pregnancy	Pilot study	31 pregnant women with type 2 diabetes	*After10 weeks of pregnancy *Intervention: until delivery
Yew 2021 ^10^	Singapore	SMART-GDM(Habit-GDM)	Improve glycemic control and maternal and neonatal outcomes in gestational diabetes mellitus (GDM) and prevent excessive gestational weight gain (EGWG) with a smartphone coaching application	RCT	*340 women -intervention group:170 -control group:170	*12-30 weeks in pregnancy with GDM *Intervention: from 27 weeks until 38 weeks of pregnancy
Sandborg 2021 ^42^	*Sweden*	*Healthy Moms*	Evaluation of the Effectiveness of the healthy mom application in the management of GWG, dietary habits, physical activity, glycemia, and insulin resistance	RCT	305 pregnant women -intervention group:152 -control group:153	*First trimester *Intervention:6 months
Jersey 2022 ^20^	Australia	Lwdp (Living Well during Pregnancy	Implementation of a tele-coaching program for reducing excessive gestational weight gain (GWG) in pregnant women at high risk.	Type II hybrid-effectiveness implementation design	*188 pregnant women -Intervention group:142 -historical comparison (Control):46	* < 20 weeks pregnant *Intervention:6 months
Whitaker 2022 ^18^	USA	INSPiRE (Increasing Steps in Pregnancy (	Assesses the feasibility, acceptability, and primary Efficacy of a remote health coaching intervention to increase physical activity and decrease sedentary behavior in pregnancy	Pilot study	34 pregnant women	*Between 8 and12 weeks of gestation *Intervention: until delivery
Steinberg 2022 ^23^	USA	Sweet Mama	Investigating the patient-related factors that are linked to the utilization of a new smartphone application designed for diabetes support and health management during pregnancy.	Pilot study	22 low-income pregnant women with GDM or T2DM	*All ages of gestation *Intervention:14 days
Lee 2023 ^37^	Korea	Aluvuu	Explore the impact of fetal images generated in VR on MFA (Maternal-fetal attachment) and depressive symptoms with the help of prenatal-coaching mobile app	RCT	88(44 people in every group)	*After 20 weeks of pregnancy *Intervention:4 weeks
Mohammadian 2023 ^15^	Iran	-	Exploring the impact of health coaching implementation on GDM prevention in overweight pregnant women	Quasi-experimental study	64 (overweight pregnant women, 32 in every group)	*12–14 pregnancy weeks. *Intervention: 8 weeks
Mattson 2023 ^24^	USA	-	Evaluation of the feasibility of telehealth nutritional counseling to address gestational weight gain	Quasi-experimental study	29 obese pregnant women	*Between12 and 27 weeks of pregnancy *Intervention:6 weeks
Jersey 2024 ^31^	Australia	-	Assessment of the effectiveness of the telephone-based coaching program(lwdp) for GDM prevention	Cost-Effectiveness Analysis	142 women (using lwdp)^20^ Compare with 1,315 overweight& obese pregnant women) routine care)	-
Téoule 2024 ^38^	Germany	Buddy Healthcare	Evaluate the effectiveness of a lifestyle intervention based on application and prenatal care program to provide patient-centered care and focus on the biopsychosocial health of women and their families	RCT	97 pregnant women *intervention group:49 *Control group:48)	* < 20 weeks (first trimester) *Intervenient: until delivery
Gibbs 2024 ^25^	USA	SPRING	Evaluation of the feasibility, acceptability, and efficacy of the sedentary behavior reduction program (SPRING intervention)	RCT	54 pregnant women -intervention group:34 -Control group:17 With advanced pregnancy outcomes (APO) and *reducing sedentary behavior (SED)*	*First trimester of pregnancy *Intervention: from14 weeks up to 38 weeks of pregnancy
Muller 2024 ^39^	France	ouge Grossesse	Assessment of mobile coaching application impact on physical activity in pregnant women	RCT	250 pregnant women (125 in every group)	*15 weeks of pregnancy *Intervention:12 week
Li 2024 ^40^	China	-	Exploring the impact of dietary intervention programs on diet quality, behavior, and nutrition literacy	RCT	88 pregnant women (44 in every group)	*6–12) < 12 weeks (of gestation *Intervention: until 24 weeks of pregnancy
Duan 2024 ^41^	China	Better pregnancy	Development and evaluation of the acceptability of the gestational diabetes mellitus application	Pilot study	100pregnant women at risk of GDM and obese	* ≤ 12 weeks of pregnancy *Intervention: one week
Pregnancy +postpartum						
Rissel 2019 ^32^	Australia	GHiP (Get Healthy in Pregnancy)	Comparing the outcomes of two GHiP (Get Healthy in Pregnancy) program service options: a telephone-based health coaching service, along with information, with an information-only option	RCT	322 pregnant women enrolled (89 completed the program,42 intervention,47 control group)	* < = 18 weeks of pregnancy until12 months after postpartum *Intervention: until 36 weeks of gestation
Bogaerts 2020 ^13^	Belgium	INTER-ACT	Development and evaluation of the INTER-ACT app	Pilot study	*In the quantitative phase: - 50 pregnant women -43 health care providers *in the quantitative phase: -Semi-structured interviews, health care provider: n = 15 - focus group: n = 2 * Healthcare providers and pregnant women in the pilot phase for user experience (n = 9	*12-42 weeks of pregnancy *Intervention time for app evaluation: 3 weeks
Coughlin 2020 ^26^	USA	H42/H4U (“Healthy for Two/Healthy for You”)	Evaluation of the acceptability of the behavioral health coaching program in pregnant women to have suitable GWG and retention weight in postpartum	RCT pilot study	26 women (any group of 13 women)	*11-16 weeks of pregnancy. *Intervention:26 weeks in pregnancy from12 to14 weeks in postpartum
Paulsamy 2021 ^43^	India	MOM	Identifying the relation between the m-health support and Maternal Observation and Motivation (MOM) program in the health of mothers & newborns	Comparative study	196 pregnant women -intervention group (study group):94 -Control group:102	*at least 20 weeks *Intervention: until 36 weeks of pregnancy
Vinay 2022 ^44^	France	Hy-Result	Assessment of the women’s experience from using the Hy-Result coaching app for self-interpretation of HMBP (home blood pressure) during and post pregnancy.	Observational study	70 pregnant women	*First trimester until three trimesters *Intervention: 7 days
Quansah 2024 ^45^	Switzerland	My Sweetheart	Examining the influence of interdisciplinary psychological and lifestyle intervention on metabolic and mental health in the prenatal and postpartum period in pregnant women with GDM	RCT	211 women with diabetes Mellitus -intervention group:105 -Control group:106	*Between 24- 32 weeks of pregnancy *Intervention: (15moths) until1year after delivery

**Table 2:** Study Technological Features

**Author/year of publication**	**Coaching practitioner**	**Coaching targets in pregnancy**	**Technology used**	**Coaching Methods/theories/app developing**	**outcomes**
Preconception +pregnancy					
Van Dijk 2016 ^34^	By system & health care professionals	nutrition, lifestyle behaviors (vegetables, fruit, alcohol, tobacco, folic acid supplement) physical activity, medication	web-based coaching platform, text messaging, and email	*Intervention group: receiving text messaging, emails (tips, recommendations, seasonal recipes, vouchers, questions about behavior, nutrition, pregnancy conditions, having MI (motivational interviews), using Bandura’s social cognitive theory (for self-efficacy, Fogg’s behavior model (for motivating to change, social influence, and the ASE model (for coaching APP development, education, and prevention	*Improving lifestyle behaviors *Having strong effectiveness and strong usability
Gootjes 2019 ^17^	By application	nutrition and lifestyle behaviors (vegetable and fruit intake, folic acid supplement use, smoking, and alcohol use)	Smartphone, web-based coaching app. SMS text messages and email	Intervention group: receiving personal coaching by text messages (SMS), email messages containing recommendations, motivation, education, seasonal recipes, and vouchers (improve self-efficacy), using the attitude, social influence, and self-efficacy model& Fogg’s behavior model using for platform content: Prochaska and DiClemente’s (readiness for behavioral change)	*Using & compliance rate was high overall *No significant Associations in smoking and alcohol use between the higher NSS (Neighborhood Status Score) & lower NSS *Women with higher NSS improved their fruit and vegetable intake and improved their lifestyles. *Improvement of lifestyle and nutrition in deprived neighborhoods, especially
van Dijk 2020 ^35^	By system & health care professionals	nutrition and lifestyle, physical activity	Smartphone, email, message, personal online webpage	*Intervention group: using Personal& motivational online coaching, including seasonal recipes, messages, incentives, feedback, recommendations, and additional questions regarding the participant’s nutrition, using a calendar in-app for reminder hospital appointments and taking folic acid, monitoring prescribed medication *Control group: not anything received	*High compliance and the larger improvements in lifestyle and nutritional behaviors& vegetable consumption, reduction of DRS (dietary risk score) in the intervention group
pregnancy					
Dodd 2017^28^	Nutritionists and research assistants	Lifestyle, nutrition, physical activity, behavioral strategies	Smartphone, coaching web app, and telephone calling	*Both groups (Lifestyle Advice Only Group and “Lifestyle Advice plus Smartphone Application Group) -Receiving lifestyle intervention including Nutrition, physical activity, behavioral strategies, Face-to-face sessions &telephone calls with a dietitian & research assistant *Intervention group (Lifestyle Advice plus Smartphone Application Group): receiving advice on lifestyle and application, using behavioral change strategies, encouragement to goal setting, self-monitoring to identify potential barriers, solve problems, and using personal development strategies	*Primary outcomes: the program has no impact on the healthy eating index (HEI), improved dietary quality across pregnancy overall, and use of the app was poor *Secondary outcomes: no significant difference between the two groups in macronutrient use. Consumption of the number of servings of dairy per day increased, consumption of noncore food groups decreased, physical activity was similar between the two groups, application helped to time exercise, and a good choice of nutrition
Jelsma 2017 ^33^	lifestyle coaches, PhD students in nutrition or human movement science, midwife, dietitian, information technology, familiar with MI (motivational interview), or have experience in an intervention study (32 hours educated about MI)	Lifestyle, diet, physical activity	mobile phone, telephone call, email, pedometer	* Intervention group: receiving face-to-face sessions, telephone coaching calls, personal lifestyle coaching, motivational interviews, using a pedometer, Dynaband, a food diary, action goal card and a manual of information	*Primary outcome: high satisfaction with the program, but not compliance with MI principles *Secondary outcome: no significant relation between the process elements & GWG (but the PA group reached less GWG) with practitioners who had ‘beginning proficiency’ in MI compared with practitioners who did not.
Willcox 2017 ^29^	Dietitian, diabetes nurse educator (Physios, Midwives, and Obstetricians)	diet, physical activity, weight	mobile devices (phone), text messages, responsive information website (viewable on mobile devices), video messages, chat room interaction via Facebook	*Intervention group: Receiving interactive messages (every week), practical advice, private Facebook chat, encouragement of positive health behavior & having goal setting, using videos created by practitioners & posting questions with practitioners and mothers& using Behavior change guidance (Social cognitive theory and the CALO-RE taxonomy), evidence-based sources such as IOM guidelines, Australian Dietary Guidelines for Pregnancy Australian Physical Activity & Sedentary Behavior Guidelines *Control group: receives standard care	*Primary outcome: feasibility) The intervention was helpful in the Intervention group ( *secondary outcome: signiﬁcantly lower GWG in the intervention group, low reductions in intensity of physical activity – frequencies of key food groups, Not were differences
Seward 2018 ^19^	Trained health coaches	Lifestyle, diet, physical activity, screen time, sleep	mobile phone, phone calls, emails, text messages , transcript devices (phone calling)	*Intervention group: delivering evidence-based lifestyle behavior, Calling by health coachee(8-20 min), using patient-centered approach motivational interviews, motivation for personal health and baby health (support, accountability), goal setting based on level of self-efficacy, readiness to change, preferences, and value& Discussing barriers, using lifestyle behavioral theories: Social Cognitive Theory, Health Belief Model, and Protection Motivation Theory, evidence-based, attention to cardiovascular healthy goals	*Primary outcome: Feasibility (enrollment & retention were good), Acceptability (High satisfaction from coaching and strong acceptability from intervention) *Secondary outcome: had a positive impact on weight management, increased motivation, accountability, and support from health coaches, and a high level of trust in coaches
Sung 2019 ^36^	endocrinologists, nurses, and nutritionists	diet, exercise, medication	mobile phone, app, Messages, glucometer Bluetooth, accelerometer, wireless network	*Intervention group: receiving messages from coaches through the mobile application including tailored medical and nutritional guidance), receiving messages from the application (twice about adequate diet and walking & communicating with health care providers, standard care, measuring physical activity level by accelerometer *Control group: standard care, common GDM management, and using mobile health care apps freely	* Improved insulin resistance * Body mass index, weight, and percentage of body fat were significantly lower in the intervention group after delivery * Significant difference in HbA1c between the two groups after delivery * No significant difference in the neonate large for gestational age and cesarean section at the time of delivery * Improve self-efficacy
Li 2019 ^16^	Diet practitioners	diet, weight	Smartphone, coaching app	*Intervention group: Receiving food coaching app, food, images uploading, receiving guidelines, and exact guidance& coaching comments from professional dietitians during the day (8 AM to 8 PM) *Control group: receiving standard food orientation in pregnancy	*Acceptance &satisfaction with the app were high *Effectiveness: cholesterol consume was less *intervention group have optimal overweight& compliance with IOM guidelines
Darrall 2020 ^30^	trained health coaches in motivational interviews	nutrition, weight, physical activity	mobile phone, pedometer (Fitbit Zip), Fitbit app, telephone calls	*Intervention group: health coaching telephone calls (20 min) with behavior change Strategies (self-determination theory, including action planning, goal setting , and self-monitoring) and motivational interview, using Australian Physical Activity and Sedentary Behavior Guidelines	*Primary outcome: syncing activity data with a mobile phone was feasible *Secondary outcome: step counts, active minutes, and GWG, no significant increase in physical activity &decreasing GWG (Due to missing pedometers, neglecting to use them, and having problems with engagement in behavior change techniques engagement,
Yee 2020 ^27^	Obstetricians, Registered Dietitians, and Certified Diabetes Educator for the development of the program	nutrition, diet	mobile phone, text messages	*Intervention group: using supportive or educational text messages about healthy foods (Health Belief Model), motivational Messages (Theory of Self-Efficacy), Technical & support Appointments, reminders, or tips for the management of diabetes (Cognitive Load Theory)	Text messaging causes Enhanced Motivation, a desire for more interaction, Reduced Social Isolation, Increased Knowledge, and improved comfort with the healthcare team.
Yew 2021 ^10^	By system	diet, Self-monitoring blood glucose (SMBG: Self-monitoring of blood glucose), physical activity, weight tracking, GDM	Smartphone, Web Bluetooth, weighing scale, gloomier linked to a smartphone, messages& manual chat Functions, interactive messaging platform, food database, glucometer, phone pedometer app, Bluetooth	*Intervention group: receiving education, and guidelines about nutrition & physical activity, Virtual coaching through application about diet, weight, SMBG, and Physical activity, sending questions to the practitioners(,(answer in 24 h at most), generic automated text message for receive healthy behavior beneficial for GDM management, educational lessons on GDM and self-management were delivered via a virtual coach, using Health Belief Model constructs to develop the app for change the behavior (common theory in pregnancy) *Control group: usual care and information, guidance on nutrition & physical activity	*Primary outcome: proportion of participants with EGWG (Excessive gestational weight gain): Not significant *Secondary outcome: absolute gestational weight gain (GWG and Better glycemic Control in mothers in the intervention group. Maternal delivery outcomes were not significant; newborn complications in the intervention group were signiﬁcantly lower
Sandborg 2021^42^	by system	diet, weight physical activity	smartphone, coaching application, text message, video, accelerometer	*Intervention group: Receiving information; push notifications and reminders, encouragement, using behavior change techniques (goal setting, shaping knowledge, feedback, and monitoring), social cognitive theory, using the guidelines of the Nordic Council of Ministers, the National Academy of Medicine *Control group: receiving standard care	*Primary outcome: -No significant effect on GWG overall (but obese women before pregnancy in the intervention group gain less weight) *Secondary outcomes: - The Swedish Healthy Eating Index was high in the intervention group -There were not significance differences on body fatness, MVPA (moderate-to-vigorous physical activity), glycemia, and insulin resistance
Jersey 2022^20^	dietitian Experienced in the delivery of prenatal services & trained in motivational interview	Nutrition, weight Physical Activity, Sedentary Behavior	Smartphone, telephone call, messages, website	*Intervention group: receiving telephone calls (60 min at first, then 30 min) with motivational interviews & health behavior coaching, goal setting, self-monitoring, identifying potential, barriers, problem-solving, identifying stimulus social support, control, mindful eating, positive self-talk, and self-reward, social cognitive theory include (self-efficacy, social support, outcome expectancies) developing skills, using lwdp, encouraging messaging, using study workbook * Control group historical comparison: (historical comparison): usual) face-to-face (dietetic care	*Primary outcomes: improvement in using and implementing the program (high satisfaction with workbook and quality of coaching) *Secondary outcomes:(effectiveness) improvement in diet quality, intake, fruit & vegetables, and weekly physical activity, and decrease in sedentary time in the intervention group; GWG was similar between the two groups
Whitaker 20222 ^18^	health-related fields, bachelor's, and were educated to use motivational interviewing approaches	physical activity, sedentary behavior	Smartphone, Zoom, telephone, text messages, Fitbit Inspire wrist monitor& Fitbit app, mobile app (Healthie), activPAL monitor	*Intervention group: conduct personalized virtual health coaching sessions by Zoom or telephone (30-45 minutes then15-30 minutes), Tip sheet, Text messages (to reinforce call contents), encourage physical activity with Fitbit, share educational materials with Healthie app, using Self Determination theory and Social Cognitive theory, motivational interview, goal setting, doing self-monitoring, measuring physical activity& sedentary time with activPAL monitor	*Primary outcome: good feasibility, high acceptability& satisfaction, primary efficacy (increases in daily steps, standing time, and decreases in total sedentary time) *Secondary outcome: improvement of physical activity by increasing daily steps & stepping times and decreasing the total sedentary time in pregnancy
Steinberg 2022 ^23^	by system	diet, physical activity, and medication management, glucose monitoring, behavioral coaching	smartphone, coaching application, text messages, video	Intervention group: receiving educational materials, using motivational content, goal-setting, self-efficacy, behavior change theory, instructional videos, reminders, and receiving feedback	*Women with gestational diabetes use more apps than women with T2DM * Longer duration of app use was related to: greater self-efficacy, more electronic health literacy, and lower patient activation
Lee 2023 ^37^	by system	Health behavior, weight, nutrition, blood pressure, glucose, activities (yoga, exercise, and stretching)	Smartphone, coaching app, virtual reality, headset, 3D ultrasounds	*Intervention group: Receive Coaching app with recommendations on health behavior, nutrition, blood pressure, weight, glucose levels, fetal ultrasound images, and a private diary service for mothers to imagine their children as they expect with the touch of a screen *Control group: Receiving coaching app without VR Images	*Primary outcome: Increase interaction with the fetus and fetal appearance perception in the intervention group (VR group) *Secondary outcome(adverse) decreasing &improvement mother depression
Mohammadian 2023 ^15^	undergraduate midwife	Nutrition, Physical activity, weight	mobile phone, telephone calls, messages	*Intervention group: receiving telephone coaching call (30-45 min), encouraging messages, coaching model (GROW (Goals, Reality, Options, and Will), using promotion of positive behavior, evidence-based information (lifestyle, Nutrition), decreasing dangerous lifestyles, weight management, problem-solving, activity planning, self-monitoring	*Primary outcome: -The incidence of GDM (Gestational Diabetes Mellitus) was not significant but low in the intervention group, and there was high satisfaction with the program *Secondary outcome: GWG (Was considerably lower in the intervention group - Baby and mother outcome not significant (in total, in the intervention group, there was less or didn’t exist:(Did not exist Preeclampsia in the intervention group, in the control group 2 case) -In total, the coaching program was effective
Mattson 2023 ^24^	Registered dietitian.	nutrition, GWG	telehealth portal, videoconferencing, Bluetooth, weight scales, social media	*Intervention group: personal coaching, encouragement, using self-monitoring, goal setting, motivational interviewing and the IOM (Institute of Medicine) guidelines for GWG	*Telehealth visits were helpful, feasible, and acceptable, *Adherence to self-weighing was high
Jersey 2024 ^31^	-	-	-	-	*The cost of lwdp was lower & had higher effectiveness than routine care. The lwdp counseling program in tests was excellent. (lwdp was cost-effective in the prevention of diabetes)
Téoule 2024 ^38^	trained midwives and medical assistants (pregnancy companions) trained with motivational interviewing strategies	physical activity, weight, physiological processes, nutrition	Smartphone, video calls Doctolib App, Buddy Healthcare app, fitness tracker wrist, chat	*Intervention group: receiving personalized virtual health-coaching sessions by Doctolib App (10 -60 min) about pregnancy worries, fears, and symptoms, partnership, diet, addiction, and financial situation, providing advice and educational materials on a healthy lifestyle, such as healthy nutrition and physical activity by Buddy Healthcare app) integrated personal support, motivational interviews, encouragement to physical activity, receiving reminders (to enter the physical activity), and standard care *Control group: standard care: visits by obstetricians and midwives based on maternal guidelines in Germany, using a fitness tracker wrist for physical activity self-monitoring, receiving the buddy health care app included (questionnaires about demographic information at enrollment and participant satisfaction without pregnancy information and chat ability)	*Primary outcome: Efficacy was good by The increasing level of physical activity decreases the rate of excessive weight gain. *Secondary outcome(exploratory): decrease EGWG (excessive gestational weight gain) in the intervention group (but not significant)
Gibbs 2024 ^25^	trained health coaches	sedentary time, physical activity (steps standing time (	smartphone, app (related to activity monitoring), videoconference, wrist wearable monitor for tracking activity and steps (Apple Watch, Fitbit), and BP, accelerometer (activPAL), Facebook	*Intervention group: receiving remote health coaching(individual/interpersonal), doing self-monitoring, tracking steps and sedentary times, using Facebook for sharing experiences, using goal setting, motivational interview, and an adjustable workstation for reduction of sedentary time *Control group: no intervention	*Decrease in sedentary time *Increasing standing * Retention was high. feasibility) *Did not increase steps *The program was useful, beneficial, and had the most useful components(acceptability)
Muller 2024 ^39^	by system (Medical experts in sports medicine, gynecology, and obstetrics, computing experts, and experts in training and coaching for the development of the app)	physical activity	smartphone, coaching app, pedometer, e-mail	*Intervention group: receiving personalized coaching, notification and feedback, encouragement, using social cognitive theory, goal setting, and self-monitoring *Control group: no intervention (Step tracking only)	*Physical activity significantly increased and Daily steps were higher in the intervention group
Li 2024 ^40^	Trained midwives	diet, GWG , gestational diabetes	Smartphone, WeChat application, video, text message	*Intervention group: doing face-to-face and online personal consultation, receiving education, encouragement, reward, reminders and feedback, nutrition booklet and models, Group communications) WeChat ( using the health literacy model and behavior change techniques) Problem-solving, Self-monitoring, social support, and action planning, doing behavioral practice/rehearsal, and conserving mental resources, using guidelines from the Chinese Dietary Guidelines for Pregnancy and the GWG range recommended by the IOM *Control group: common care (check-ups and Monitoring)	*Improving in: -eating behavior, -nutrition literacy and -quality of nutrition and gestational weight, *Don’t have significant results in diabetes results
Duan 2024 ^41^	Nurse (Software engineers, researchers, User Interface (UI) designers for app development)	diet, exercise, weight, blood sugar, blood pressure	mobile phone, coaching application, text message, video	*Intervention group: Receiving personalized coaching, education, and feedback Improving self-efficacy, giving a reward, receiving reminders, having family support, using behavior change techniques (health belief model, goal setting), and evidence-based information such as articles and the Australian JBI evidence pre-classiﬁcation and recommendation level system	*Acceptability of the app was good, and it can be a better help for the prevention of GDM
Pregnancy +postpartum					
Rissel 2019 ^32^	university-qualified coaches (dieticians, exercise physiologists, midwives)	gestational weight gain, nutrition, physical activity	Telephone calls	*Two groups: Receiving Evidence-based written resources& a booklet about weight gain, nutrition, and physical activity in pregnancy *Intervention group: receiving information& telephone-based coaching call in pregnancy (15-25 min) with behavioral change techniques *Control group: only information& one telephone calling(20-30min)	*Improvement in GWG in the intervention group (but not significant) *The program was useful and supportive, and facilitated conversations with mothers *Decreasing intake of soft drinks daily & weekly takeaway in the intervention group. *In both arms, there were positive changes in total. * The program could not perform an effectiveness evaluation in the rate of program left, low uptake& retention
Bogaerts 2020 ^13^	general practitioners, gynecologists, midwives, dieticians, and user-experience researchers for app development and coaching	weight nutrition, physical activity, mental wellbeing- (sleep, stress, emotional)	smart phone, coaching app, Bluetooth, activity tracker, weighing scale, text message	*Coaching app development method: conducting semi-structured interviews, focus groups, and surveys for app development. Applying nutritional recommendations of the Superior Health Council of Belgium, gestational weight gain guidelines from the Institute of Medicine (IOM), and guidelines of the Flemish Institute for Healthy Living, motivational interviewing techniques, goal setting, positive messaging, Tips for encouraging weight and physical activity, mental well-being, and healthy eating	*Point of view of health care practitioners and pregnant women about coaching app development: -Application can increase a healthy lifestyle in pregnancy and postpartum -pregnant women said that the app should have a graphical display, a weekly notification, Support messages based on goals - app increased awareness -The combination of face-to-face coaching and the app was valuable
Coughlin 2020 ^26^	master's degrees had strong backgrounds in behavioral health coaching, nutrition, or maternal health, trained in motivational interviewing	weight, physical activity, wellness (stress, sleep, mood)	mobile Phone &app, telephone calls, Lose It app	*Intervention group: -In pregnancy: patient-centric coaching calls (20-30min, weekly), receiving reminders, learning activities (COACH model) -in postpartum: coaching calls, learning activities with behavior change Strategies, individualized goal setting tracking diet, weight, and physical activity (Lose It app) *Control group: receiving health education session (nutrition, low-cost, healthy shopping, food, and general prenatal safety(45min))	Primary outcome: feasibility (coaching calls, learning activity, self-monitoring frequency)
Paulsamy 2021 ^43^	master's degree in Nursing in Obstetrics and Gynecology, master's in Mental Health Nursing	Supplement nutrition, diet habits, physical activity, mother’s wellbeing	smartphones, Fit app, WhatsApp, message, call, electronic scales	*Intervention: Face-to-face individual education visits with Encouragement and awareness & motivational counseling (about nutrition, supplemental medications, well-being, physical activity), receiving messaging by WhatsApp, virtual health coaching sessions, measuring physical activity by a fitness app, applying custom-made interactions, and routine care *Control group: receiving standard prenatal care and routine protocols	*Maternal outcome: improvement in the: -anemia - increase in weight - physical activity& mother's wellbeing *Newborn outcome: -signiﬁcantly improve on Birth weight, crown–heel length
Postel-Vinay 2022 ^44^	Directly by the system and indirectly by the Physician, midwife	Blood pressure	Smartphone, tablet, mobile coaching application, text messages, email, sphygmomanometer	*Intervention group: Measuring BP by patients at home & reporting results with a medical condition in the online form, receiving automated interpretation& messages from the app, sharing blood pressure results in PDF format (graphics + text messages) by email to practitioners, & receiving advice from the app, consulting with a practitioner	*According to most mothers’ reports: - the program helps them when consulting with physicians, -was easy to use and reliable, -had effectively monitored BP, -color code classification was “clear, -app was helpful (62%), very few said it was worrying (7%)
Quansah 2024 ^45^	physician, diabetes specialist nurse, trained psychologist, dietician, physiotherapist, midwife	Diet, physical activity, and mental health, social support, gestational weight gain (GWG), postpartum weight retention	Phone, telemedicine platform	*Intervention group: Receiving motivational Interviewing, encouragement, recommendations , using the Health Action Process Approach model , strengthen self-efficacy, providing social support, holding group workshops, Using evidence-based guidelines, the American Diabetes Association and The Endocrine Society, National Institute for Care and Health Excellence (NICE) guidelines *Control group: common care (education and recommendations about diabetes and its control, healthy lifestyle, consultation with a dietitian and follow up and clinical visits)	*Primary outcome: -not enough evidence to decrease the weight and depression symptoms *Secondary outcome: -Increased body mass without fat - decreasing in GWG - A higher proportion of women were without weight maintenance at one year postpartum *Overall improvement in metabolic and mental measures, but it was not significant

###  Inclusion criteria

 This review included English-language studies on the development, implementation, or evaluation of digital health coaching interventions during pregnancy. Interventions were delivered via apps, calls, messages, or other digital platforms. Studies applying coaching methods (e.g., goal-setting, motivational interviewing, active listening) were included even without explicit “coaching” terminology. Interventions were limited to those occurring exclusively during pregnancy, initiated before pregnancy and continued into pregnancy, or started during pregnancy and extended postpartum. Pregnant women of any age or medical condition were included.

###  Exclusion criteria

 Studies were excluded if they were non-English; focused solely on the development of coaching interventions without implementation or participant outcome evaluation; limited exclusively to the preconception or postpartum periods without including the pregnancy stage; lacked interactive health coaching components; involved the delivery of health coaching without digital technology; or were non-original, non-peer-reviewed publications (e.g., reviews, protocols, letters, conference abstracts) or lacked a full text.

## Results

 The total number of articles obtained from the search in databases was1827; after removing 667 duplicates,1160 were left for screening, and1099 papers were excluded based on inclusion and exclusion criteria in the title and title-abstract screening. Out of a pool of 61 articles, 30 were excluded based on specific criteria. These included two articles without digital coaching,14 focused exclusively on pre-gestation or postpartum, and14 conducted without coaching. Finally, 31 studies were included in our review. The study selection process is illustrated in the PRISMA-SCR flow diagramin Figure 1. The summary of the included papers and characteristics of digital health coaching interventions in pregnancy are shown in Tables 1 and 2.

 The included studies were published in the period from 2016 to 2024; most of them were conducted in the United States of America (USA) (23%),^18,19,23-27^ and Australia (19%).^20,28-32^ Figure 2 shows the countries and the year of study publication. In this figure, a study by Jelsma et al. (2017) titled Europe on the Map, nine countries, including Austria, Belgium, Denmark, Ireland, Italy, the Netherlands, Poland, Spain, and the United Kingdom (UK), participated.^33^

**Figure 2:**
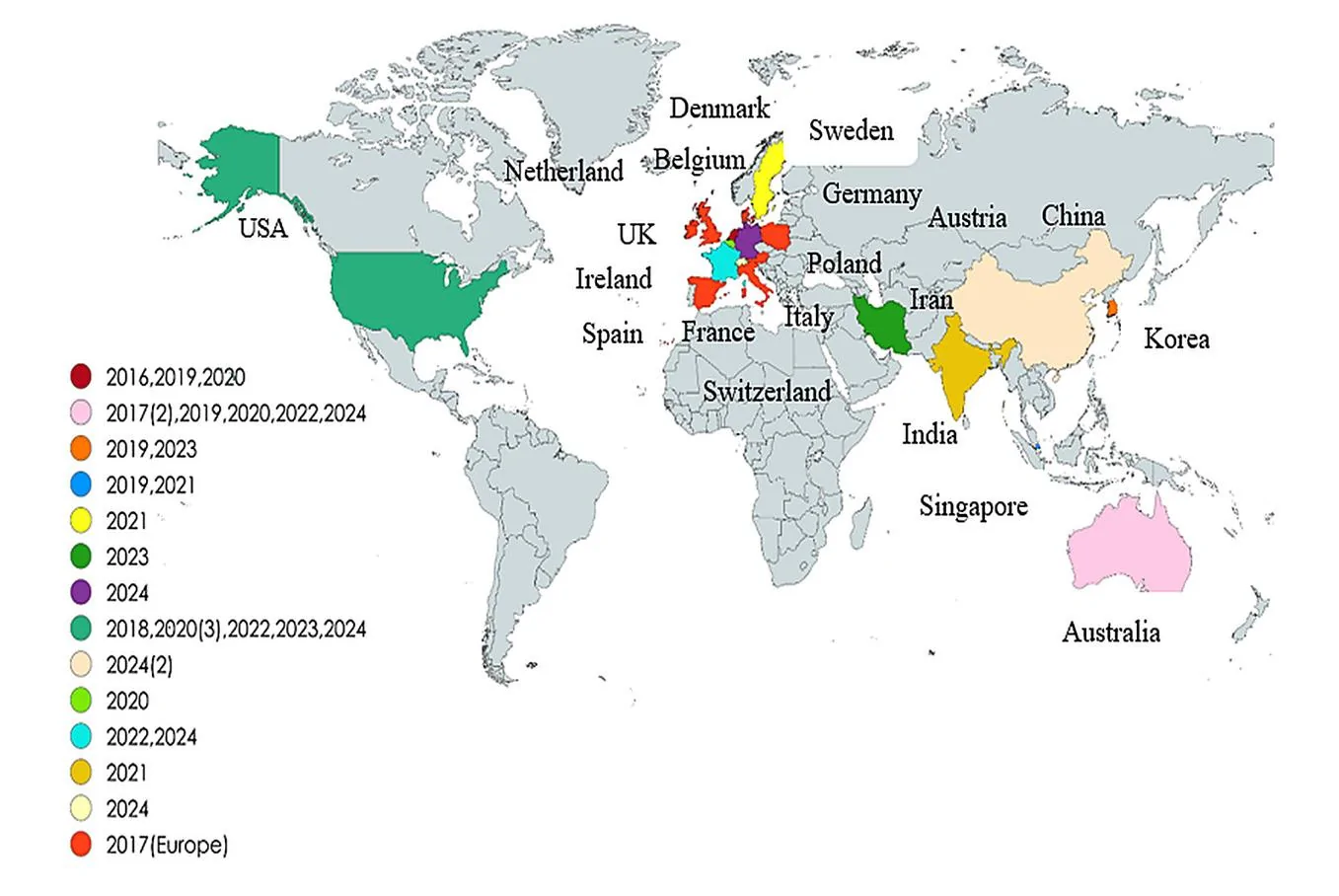
The countries conducting the studies and the year of their publication

 Three studies (10%) were related to pre-pregnancy and pregnancy,^17,34,35^ 22 studies (71%) were done in pregnancy,^10,15,16,18-20,23-25,27-31,33,36-42^ and 6,(19%) were related to pregnancy and postpartum;^13,26,32,43-45^ the design of all studies mainly was Randomized Control Trial (RCT) (71%), as shown in Figure 3.^10,13,16,18,19,23,25-30,32,35-42,45^

**Figure 3:**
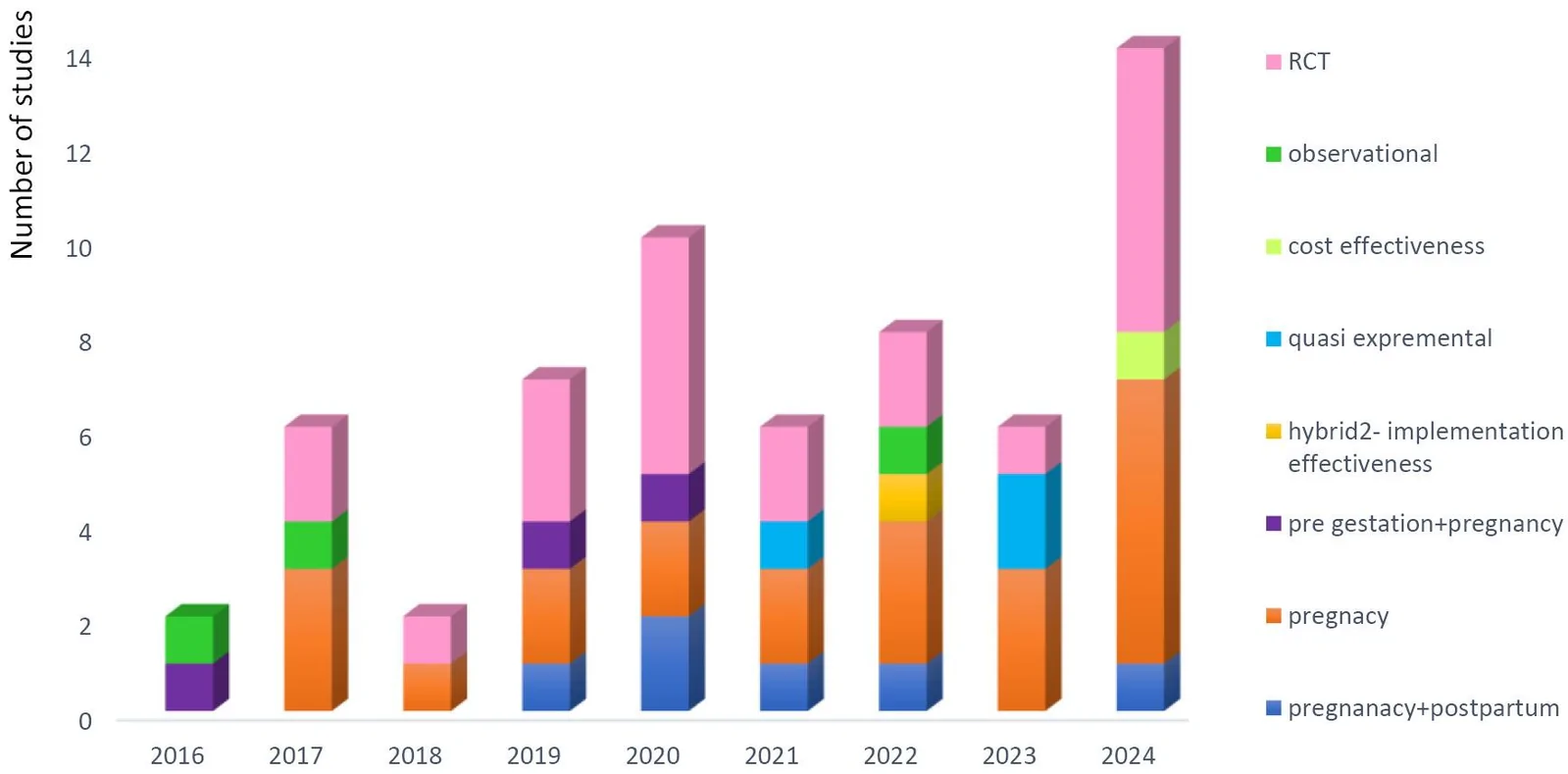
Number, Design of studies, and pregnancy periods

Figure 3 shows that our study examined other studies, including observational studies (4),

 cost-effectiveness studies (1), quasi-experimental studies (3), and hybrid-implementation effectiveness studies (1).

###  Coaching objective/target fields 

 The included studies, which addressed the development, implementation, or evaluation of effectiveness (including cost-effectiveness,^31^ usability, accessibility, compliance, feasibility, and user experience) of coaching interventions,^10,13,15-20,23-30,32-45^ focused on three main fields, which we categorized as follows:

1. Promoting lifestyle modification which included: (i) nutrition and dietary habits (e.g., eating vegetables, fruits, folic acid, supplements, and medication), examined in 25 articles (83%);^10,13,15-17,19,20,23,24,27-30,32-38,40-43,45^ (ii) Physical activity (including exercise, yoga, and reducing sedentary behaviors) examined in 24 articles (80 %);^10,13,15,18-20,23,25,26,28-30,32-39,41-43,45^ (iii) behavioral strategies related to alcohol use and smoking, were discussed in 3 papers (10%).^17,28,34^
2. Management of clinical and anthropometric outcomes including weight/gestational weight gain (GWG), postpartum weight retention, gestational diabetes mellitus (GDM), type 2 diabetes, and control of blood glucose and pressure were mentioned in 23 studies (76%).^10,13,15,16,19,20,23,24,26,27,29-33,36-38,40-42,44,45^
3. Improving maternal physiological, mental, well-being and perinatal health outcomes including stress, emotions, motivation, depressive symptoms, sleep, screen time and health literacy and maternal and newborn health/complications reviewed in11 studies (36%).^10,13,15,19,26,27,37,38,40,43,45^

###  Sample size and intervention time

 Most of the studies had a large sample size (13 (42%)),^10,17,20,28,31-35,39,42,43,45^ followed by medium (9 (29%))^13,15,25,29,37,38,40,41,44^ and small (9 (29%)) groups.^16,18,19,23,24,26,27,30,36^ In terms of intervention duration, most studies)17(57%) were related to medium-term interventions,^15-17,19,20,28,30,32-36,38-40,42,43^ 7(23%) studies had long-term interventions,^10,18,25-27,29,45^ and short-term interventions were in 6 (20%) of studies.^13,23,24,37,41,44^ See Table 3.

**Table 3:** sample size &intervention duration

**Sample size**		**Intervention duration**	
Small (< 50)	9(29%)	Short- term(< 8weeks)	6(20%)
Medium (50-100)	9(29%)	Medium- term(8-24weeks)	17(57%)
Large (> 100)	13(42%)	Long- term(> 24weeks)	7(23%)

 Some of the participants had different conditions or were at risk, including overweight and obese or high gestational weight gain (GWG)^15,16,19,20,23,24,26,29-31,33,36,41^ and diabetes.^10,16,23,27,45^ Some participants were preparing for pregnancy or enrolled with their partners;^17,34,35^ some were from deprived neighborhoods,^17^ low income,^23,27^ and with advanced pregnancy outcomes and reduced sedentary behaviors,^25^ which were in different periods of pregnancy and participated in the intervention or control groups according to the design and objectives of the studies and coaching interventions.^13,18,28,32,37-40,42-44^

###  Technology used

 The word cloud (Figure 4) illustrates the different devices and technology methods used to implement digital health coaching interventions during pregnancy. Technologies used include using smartphones,^10,13,16-18,20,23,25,28,35,37-40,42-44^ mobile phones,^15,19,26,27,30,36,41^ or mobile devices (tablets), telehealth or telemedicine portals and web-based platforms,^24,29,34,44,45^ transcript devices,^19^ a personal webpage,^35^ responsive information websites,^29^ and coaching programs or applications such as Pregnant Faster, Doctolib, Buddy Health Care, Healthie, Healthy Moms apps,^10,13,16-18,23,28,34-39,41,42,44^ and other mobile applications like Fitbit, Bit Snap, and Lose It.^10,18,25,26,30,43^

**Figure 4:**
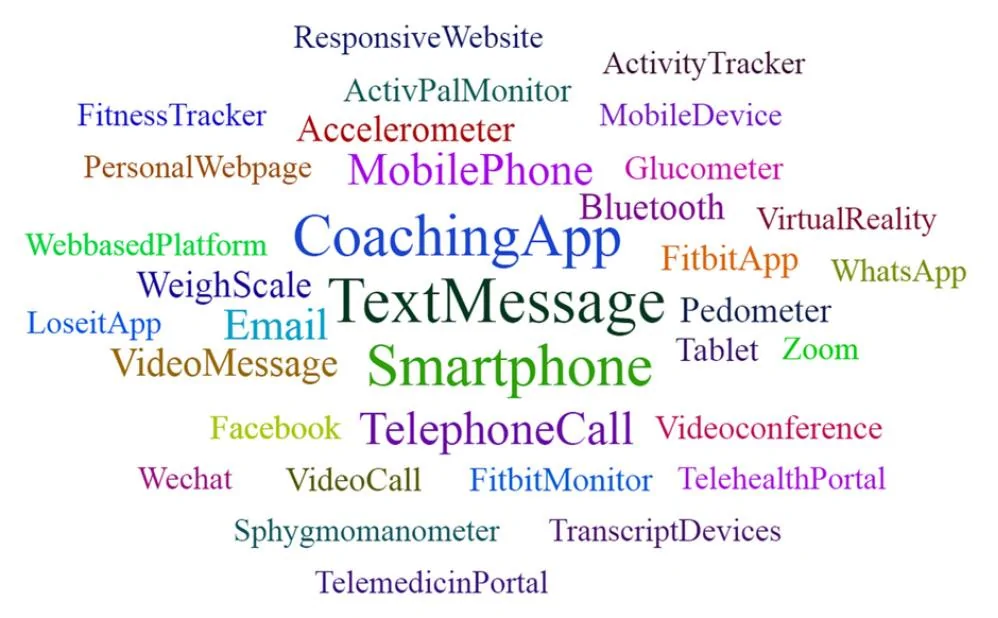
Word Cloud for Types of Used Technologies in Digital Health Coaching Interventions in Pregnancy

 Studies also indicated the use of devices for monitoring and tracking participants, including sphygmomanometer, glucometer, activity monitor (ActivePAL), Fitbit monitor, accelerometer, pedometer, fitness tracker, and weigh scale that could be linked with mobile applications through Bluetooth.^10,13,18,24,25,30,33,36,38,39,42-44^

 In these interventions, the use of telephone and video calls, text messages, reminders, email, chat, video messages, virtual reality, applying telehealth and telemedicine portals, and social platforms such as WhatsApp, Zoom, Facebook, and WeChat were also referred to as technologies to help the coaching interventions implement.^10,13,15-20,23-30,32-45^ In this figure, the technologies that were used the most in the studies, are shown in bolder and larger font, whereas the ones used less are shown in smaller font. The most used technology device was smartphone (57%), and the most technological methods for providing coaching services were text messages (66%), coaching applications (56%), and telephone calls (40%). Some studies also did not mention the type of device used for telephone calls.^32,33^ (see Tables 1 and 2)

###  Coaches’ expertise and skills

 In 7 studies (23%), coaching services have been provided only by apps or systems. ^10,17,23,27,37,39,42^ The remaining studies utilized various approaches or technologies to facilitate the coaching process alongside a human coach.^13,15,16,18-20,24-26,28-30,32-36,38,40,41,43-45^

 Multidisciplinary healthcare specialists were involved in developing coaching interventions or delivery services. Nutritionists/dietitians, PhD students in nutrition, and culturally congruent dietitians participated in 40 % of papers.^13,16,20,24,26-29,32,33,36,45^ Gynecologists or obstetricians, midwives, or undergraduate midwives involved in 40%.^13,15,27,29,32,33,38-40,43-45^ Other specialists included general practitioners,^13^ nurses, diabetes nurse educators and specialists, mental health nurses with master’s degree in obstetrics and gynecology, ^29,36,41,43,45^ Endocrinologists,^36^ information technology specialists and user-experience researchers,^33,41^ computing experts,^39^ human movement science experts and physical therapists,^29,33,45^ certified diabetes educators^27^ medical assistants (pregnancy companions), exercise physiologists and sports medicine specialists,^32,39^ and psychologists.^45^ These practitioners have some skills in delivering coaching services. They were trained with motivational interview (MI) strategies in 26% of studies, which was the most behavior modification skill that the coaches were familiar with.^18,20,26,30,33,38^ Also, practitioners have experience in the intervention study,^33^ delivery of prenatal services ormaternal health,^20^ training and behavioral health coaching,^26,39^ and health-related fields, and some were trained health coaches, though their specific expertise and healthcare fields were not mentioned.^18,19,25,34,35^

###  Coaching Methods and Theories

 In addition to receiving standard care, which includes routine prenatal protocols, brochures, and access to general information and websites, the intervention group in 97% of the studies received additional support. This support included information and educational components, supportive interactions, feedback, comments, encouragement. Specifically, participants received advice and plans tailored to their nutrition, physical activity, pregnancy conditions (such as GWG, diabetes, mental health, etc.), and health behaviors. They also used tracking systems and coaching applications. Support was delivered through personal coaching sessions, either virtual or face-to-face, via telephone, video calls, video conferences, or chat platforms (Facebook, WhatsApp, Zoom, WeChat), as well as through text messages and emails. Also they received tips, seasonal recipes, vouchers, reminders, and rewards to motivate interaction and engagement.^10,15-20,23-30,32-45^ Additionally, group sessions and social activities (workshops and sharing experiences on social media) were also done in coaching interventions.^25,40,45^

 These educational components were based on evidence-based information from articles and guidelines of organizations provided by various organizations. Such organizations include the Institute of Medicine (IOM), the Australian Dietary Guidelines for Pregnancy, the Australian Physical Activity & Sedentary Behavior Guidelines, the Nordic Council of Ministers for nutrition, National Academy of Medicine (NAM), the Superior Health Council of Belgium, guidelines of the Flemish Institute for Healthy Living, maternal guidelines in Germany, Chinese dietary guidelines for pregnancy and the GWG, Australian JBI evidence pre-classiﬁcation and recommendation level system, American Diabetes Association and the Endocrine Society, National Institute for Care and health Excellence (NICE) guidelines in 40% of studies.^13,15,19,24,29,30,32,38,40-42,45^ Also, 43% of the interventions depicted physical activity, weight, sleep, stress, blood sugar, blood pressure, and other required measures monitored by wearable devices and trackers that connected to mobile phones by Bluetooth (mentioned in the technology used segment) and mobile applications like the Fitbit and Lose it app.^10,18,24-26,30,33,36,38,39,42-44^

 Coaching interventions based on behavioral change strategies include motivational interviewing in 47% of included papers;^13,18-20,24-26,30,33-35,38,43,45^ social cognitive theory (Bandura’s) in 23%,^18-20,29,34,39,42^ and Goal setting in 47% as well.^13,18-20,23-26,28-30,39,41,42^ Other theoretical models applied in the studies included the Coventry, Aberdeen, and London – refined (CALO-RE) taxonomy,^29^ Fogg’s behavior model,^17,34^ self-determination theory,^18,30^ attitude–social influence–self-efficacy (ASE) model and self-efficacy theory,^17,19,20,23,27,34,45^ health belief model,^10,19,27,41^ protection motivation theory,^19^ cognitive load theory,^27^ transtheoretical model (Prochaska and DiClemente’s),^17^ goals, reality, options, and will (GROW) model,^15^ learning activities (connect, outcome, awareness, course, highlights (COACH) model),^26^ health literacy model,^40^ health action process approach (HAPA) model.^45^ In addition, coaching interventions employ self-monitoring techniques to identify possible obstacles, resolve issues, and implement strategies for personal development. Several interventions have provided social support, positive self-talk, mindful eating, self-rewarding, activity planning, developing skills, shaping knowledge, and doing behavioral practice/rehearsal, and conserving mental resources. ^15,18,20,24,25,28,30,31,38-40,42,45^

 The control group received standard care, which included face-to-face, telephone calls or messages, handouts, guidance, or information. They were also provided with links to information websites about pregnancy lifestyle, nutrition, physical activity, weight gain, postpartum weight loss, and follow-up measurements and tracking, or could use mobile health care apps freely without coaching interaction and services that the intervention group received.^10,15,16,20,25,26,28-32,35-40,42,43,45^

###  Outcomes 

 Out of 31 studies,14 (45%) highlighted the acceptability, high satisfaction, and compliance with coaching interventions,^15-20,24,25,32-35,41,44^ 11 (35%) demonstrated successful feasibility andusability.^18-20,24-26,29,30,32,34,44^ Additionally,21 studies (68%) demonstrated the effectiveness and efficacy of coaching interventions in lifestyle behaviors such as diet quality, decreasing sedentary times, increasing knowledge, increasing motivation, increasing accountability and support from health coaches, and enhancing interaction with the fetus.^13,15-20,25,27,28,32,34-40,42-44^

 One study found that coaching program were both less expensive and more cost-effective than routine care.^31^ Thirteen papers (42%) focused on weight management, GWG, excessive gestational weight gain (EGWG), monitoring blood pressure, and glycemic control, ^10,15,19,29,32,38,40,42,44,45^ as well as body mass index and percentage of body fat.^16,19,36^. Also, 3 studies (10%) examined maternal and newborn health, such as anemia, birth weight, crown-heel length, and depression symptoms.^10,37,43^ One study mentioned insulin resistance and glycated hemoglobin (HbA1c) levels.^36^ These results were referred to as primary or secondary outcomes.

 One study referred to programs that were not in compliance with motivational interviewing principles.^33^ However, some studies concluded that there was no significant relation between the intervention and control groups for outcomes, including EGWG,^10,38^ healthy eating index, macronutrient use,^28^ neonate large for gestational age, and cesarean section at the time of delivery,^36^ delivery outcome,^10^ depression, physical activity, decrease in GWG,^20,30,32,33,42,45^ glycemia, insulin resistance, GDM, maternal and neonatal outcome (preeclampsia).^15,40,42^ In some cases, however, overall improvement was achieved.^10,15,28,32,33,38,45^ One of the studies showed that longer duration of using the coaching app was related to greater self-efficacy, higher electronic health literacy, and lower patient activation.^23^

 The main results of the studies as a framework are visualized in Figure 5, which, based on the findings, shows the main features of developing and using digital health coaching interventions in pregnancy.

**Figure 5:**
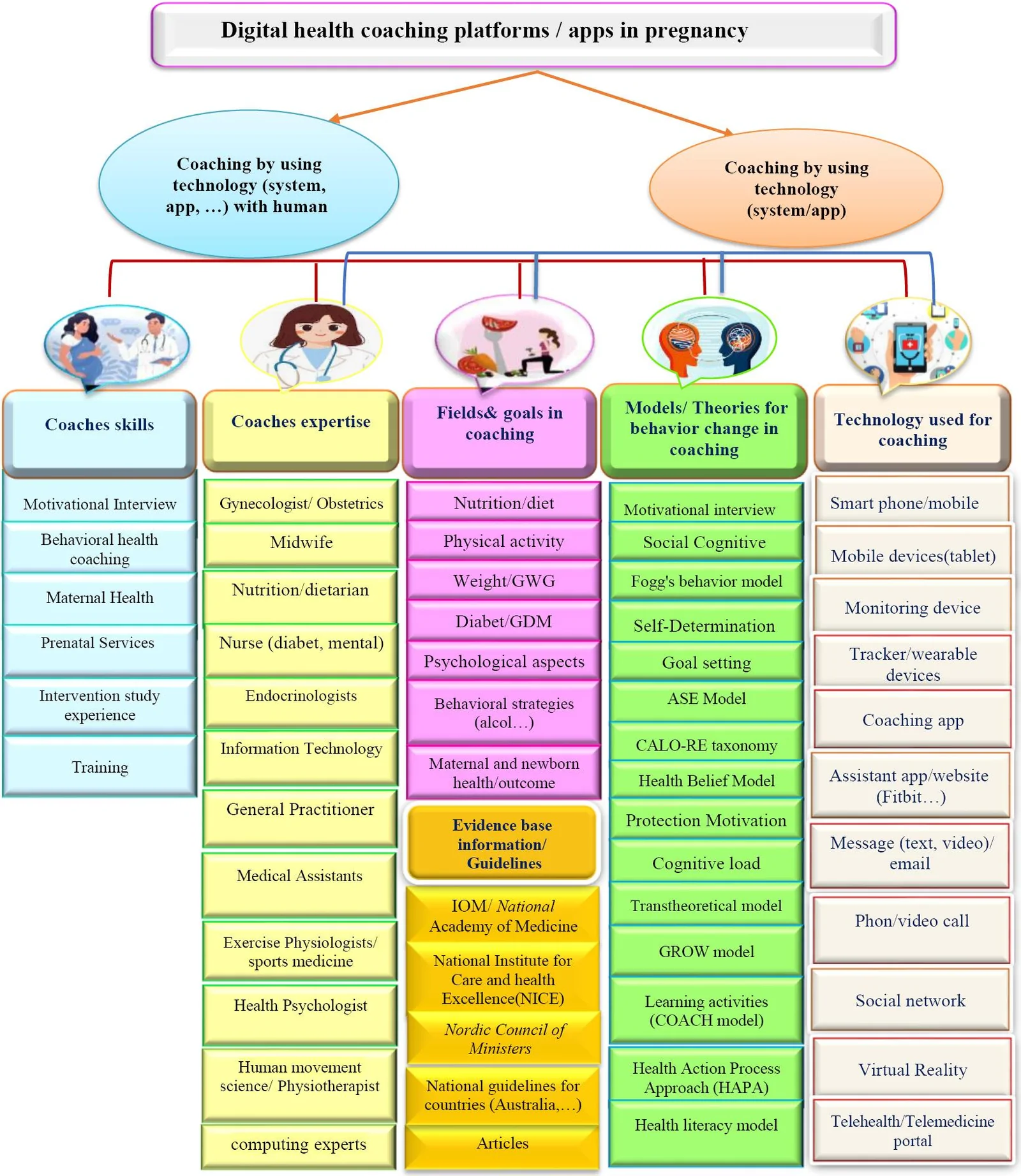
Framework for the development and implementation of digital health coaching interventions in pregnancy

## Discussion

 The primary focus of this review was on the features and requirements of digital health coaching interventions, which are regarded as innovative support models in recent pregnancy care. The results showed that most of the interventions were related to pregnancy rather than pre-pregnancy and postpartum.^10,15,16,18-20,23-25,27-31,33,36-42^ Our findings highlight the importance of pregnancy care for maternal and newborn health within the scope of the included studies. This is consistent with the recommendations issued by the World Health Organization (WHO) for pregnant women, which recommend evidence-based and timely antenatal care to optimize outcomes for both mothers and babies.^46^ Therefore, specialists should pay enough attention to the sensitivity of this period, because this time is characterized by significant physical, mental, and social changes that can influence the health of both the mother and the child.^47^

 Also, results of this review demonstrated that nutrition, physical activity, weight management and diabetes were the most targeted areas that positively impacted maternal health. However, psychological and mental aspects have been less addressed.^10,13,15,19,26,27,37,38,40,43,45^ These findings are according to Raab’s^48^ study, which reviewed app-based interventions in managing gestational weight gain and gestational diabetes and highlighted the necessity of psychological support. Although pregnancy is a critical period for the recurrence of mental health disorders, the emotional well-being of pregnant women is often overlooked in the field of obstetrics. Studies have demonstrated that when a pregnant mother experiences depression, anxiety, or stress, it raises the likelihood that her child will experience various adverse consequences, such as emotional difficulties, symptoms of attention deficit hyperactivity disorder (ADHD), and impaired cognitive growth.^49,50^

 Various technological devices and approaches are applied in coaching interventions, including smartphones, coaching apps, text messaging, and telephone calls. they are mainly aimed at improving lifestyle behaviors during pregnancy using personalized coaching. Some studies demonstrated that these technologies led to significant improvements in lifestyle behaviors, particularly in dietary habits and physical activities, engagement, and adherence to healthcare.^19,35,38,39,51,52^ Yousuf et al^53^ found that e-coaching tools reduced the risk of cardiovascular diseases through a web-based personalized intervention, which highlighted the effectiveness of digital platforms in lifestyle modification, but some included studies declared that technology may have a limited impact on some outcomes like the healthy eating index (HEI), macronutrient intake, or gestational weight and diabetes management.^28,30,36^ This suggests that technology alone may not be sufficient without the effective involvement of users or perhaps tailored design multidimensional support. The WHO^54^ in its GlobalStrategy on Digital Health, emphasizes that when developing national digital health strategies, attention should be given to elements such as an adaptable health workforce, flexible digital health infrastructure, a people-centered approach, and services and applications for scaling up that can support equitable access, user engagement, trust, and meaningful impact.

 Most of the health practitioners in our study were nutritionists/dietitians, gynecologists, and midwives.^13,15,16,20,24,26-29,32,33,36,38-40,44,45^ Additionally, most studies included a diverse team of professionals, highlighting the significance of a comprehensive and collaborative approach. ^13,26,27,29,32,33,36,38,39,41,43-45^ This approach addresses the varied needs of pregnant women and incorporates multiple areas of expertise, ultimately leading to improved outcomes.^55,56^ The impact of health coaches in providing coaching services during pregnancy can be observed through participants’ opinions.^13,19,32^ Also, Ju &Kang^57^ found that incorporating digital platforms with human coaching can have long-term effects on primary health care.

 Maternal and fetal health heavily rely on behavior change, and various theories and models, including motivational interviews, goal setting, and cognitive behavior theory, were frequently utilized in studies.^13,18-20,23-26,28-30,33-35,38,39,41-43,45^ Merten et al^58^ evaluated the effectiveness of lifestyle coaching in GWG and postnatal weight loss and demonstrated that self-monitoring of behavior, feedback on performance, and goal setting were the most common techniques for decreasing GWG.Other studies depicted the effectiveness of these theories in reducing smoking and alcohol consumption, managing weight, motivation to adhere to medication recommendations, and translating motivation into actionable steps that enhance patient self-efficacy and help to change behavior.^59-62^ Meanwhile, the motivational interview proved to be the most effective skill for practitioners, encouraging pregnant women to prioritize their health and their baby’s health through support, accountability, and readiness to change.^18,20,26,30,33,38^ This approach fosters intrinsic motivation to explore individuals’ objectives and establish Specific, Measurable, Attainable, Relevant, and Time-bound (SMART) goals.^62,63^ However, the long-term effectiveness of behavior change techniques is not guaranteed. Studies have found that patient motivation can decline significantly over time, as observed in a lifestyle program for heart disease and diabetes.^64^ Moreover, users often show decreased participation in behavior change interventions and chose easier programs, which undermines long-term adherence.^65^ Some findings explicitly state that despite the willingness and satisfaction to do digital coaching, its impact on physical activity and eating habits was less over time.^29,33^ This shows that satisfaction does not guarantee final effectiveness.^66^ However, digital tools can cause the disengagement of patients, incorrect use of applications, and lack of active participation, which decreases the effectiveness of interventions.^67^ Therefore, to achieve optimal health outcomes and sustained effectiveness, it is crucial to emphasize appropriate interventions, flexible templates, continuous support, and compatibility with user needs. These measures can enhance user adherence, participation, and interaction with digital coaching interventions. Although many studies have demonstrated coaching interventions’ efficacy and positive impact, some suggest that their effectiveness can differ based on the specific techniques used. This calls for the need to examine optimal strategies.^68^ Non-significance of results in some studies can be attributed to factors such as low compliance with programs, small sample sizes, short intervention periods, poor use of applications, missing or neglecting to use the tracker, having problems with behavior change techniques engagement, withdrawal of individuals from the study and lack of intervention on several behavioral aspects.^28,30,33,36,45^ This issue can affect the outcomes of the coaching interventions, and measures should be taken to reduce the effects of such factors in implementing coaching interventions.

## Conclusion

 This scoping review provides a framework for supporting expectant mothers through personalized content and coaching, technology integration, evidence-based practices, real-time feedback, and a holistic approach to their health during different stages of pregnancy. By considering the sensitive nature of pregnancy and providing comprehensive support, and ensuring the accessibility and effectiveness of digital coaching interventions, robust design and implementation of such systems can play a valuable role in improving maternal and fetal health outcomes. Fostering community, holding women accountable, and engaging them over time can ultimately motivate women to take charge of their health during this critical period; these strategies have the potential to promotehealthier families and communities. Further research is recommended to explore the dimensions of mental and psychological well-being, behavioral approaches of expectant mothers (such as smoking and alcohol consumption), and considering socio-economic factors in the development and execution of digital health coaching interventions.

## Limitations

 The choice of English-language studies and the exclusion of studies published in other languages may have led to linguistic bias. In addition, the included studies employed a variety of technologies and coaching methods focusing on different aspects of lifestyle. This diversity limited comparability between studies and prevented consistent conclusions on the effectiveness of the interventions. Furthermore, the inclusion of interventions extending beyond the preconception and postpartum periods made it difficult to isolate the specific effects of coaching during pregnancy.

## Competing Interests

 The authors declare that they have no competing interests.

## Data Availability Statement

 The information supporting the conclusions of this article is included within the article.

## Ethical Approval

 This work is part of a Ph. D. thesis approved by the Tabriz University of Medical Sciences (Number: IR.TBZMED.REC.1403.229). Informed consent did not apply to our study.
